# Excellent response to asfotase alfa treatment in an adolescent patient with hypophosphatasia

**DOI:** 10.1002/jmd2.12198

**Published:** 2021-02-03

**Authors:** Olivia Sarah Strandbech, Allan Lund, Elsebet Ostergaard

**Affiliations:** ^1^ Department of Clinical Genetics Copenhagen University Hospital Copenhagen Denmark; ^2^ Centre Inherited Metabolic Diseases, Departments of Clinical Genetics and Pediatrics Copenhagen University Hospital Copenhagen Denmark

**Keywords:** asfotase alfa, childhood hypophosphatasia, hypophosphatasia, skeletal pain

## Abstract

Hypophosphatasia (HPP) is a rare inherited metabolic disorder characterized by deficient activity of alkaline phosphatase, causing defective mineralization of bones and teeth. The symptoms vary from no symptoms to stillbirth or skeletal manifestations. Since 2015, asfotase alfa, an enzyme replacement treatment, has been approved for pediatric use in some jurisdictions. We describe the clinical outcome of asfotase alfa therapy in an adolescent patient with childhood HPP. The patient was diagnosed with HPP at 13 months. She had a history of hypertonia and failure to thrive from age 3 months. During childhood the patient experienced chronic skeletal pain, requiring daily use of analgesics and school absences. Her plasma pyridoxal‐5‐phosphate was elevated at >2500 mmol/L, phosphoethanolamine at 11 μM, and ALP decreased at 25 U/L. On the visual analog scale (VAS), a scale used to determine pain intensity, she stated an average of 7 (maximum 10) at age 13. She had no abnormalities on radiography. At age 13 the patient was started on asfotase alfa 1 mg/kg given subcutaneously 6 times weekly. Three months after treatment the patient had a decreased P‐pyridoxal‐5‐phosphate level of 41 mmol/L, used fewer analgesics, and a lower average VAS‐score. At every follow‐up, she continued to exhibit improved biochemical values, along with lower VAS‐scores. In conclusion, asfotase alfa significantly improved the patient's quality of life. This case suggests an association between children with HPP without radiographic abnormalities, but a debilitating pain phenotype, and a significant pain reduction on enzyme replacement therapy. Thus, this therapy should be considered in such patients.

## 1 INTRODUCTION

Hypophosphatasia (OMIM #241510) (HPP) is a rare inherited metabolic disorder caused by pathogenic variants in the *ALPL* gene which encodes the tissue‐nonspecific isoenzyme of alkaline phosphatase (TNSALP, EC # 3.1.3.1).^1^ As of March 2020, 409 different pathogenic variants, mainly missense variants, have been reported worldwide (*ALPL* mutation database: http://www.sesep.uvsq.fr/03_hypo_mutations.php). The variants are thought to affect various steps of both the folding and transportation through the secretory pathway of the TNSALP protein causing numerous malfunctions of the protein.^1^ As a result, TNSALP activity is deficient causing increased levels of substrates resulting in defective mineralization of bones and teeth.^1^ Bone mineralization is initiated inside matrix vesicles and is divided into two stages; the first stage is the production of hydroxyapatite (HA) from Ca^2+^ and extracellular P_i_. P_i_ is provided by TNSALP through dephosphorylation of substrates such as PPi, PLP, and PEA.^2^ In the second stage, HA is released to the extracellular matrix. Here, the extracellular balance of substrates/P_i_ is of great importance as increased levels of substrates inhibit the propagation of HA,^2^ explaining the defective bone mineralization in HPP.

HPP can be divided into six clinical subtypes based on the age of onset and severity of features; however, the subtypes often show overlap (Table 1). All subtypes share the same clinical hallmarks: defective mineralization of bones and/or teeth and reduced activity of TNSALP. Due to the highly variable symptoms, there is a significant difference in prognosis within the subtypes. Both the perinatal and infantile subtypes are associated with high mortality in the first 5 years of life. The two strongest indicators for a poor prognosis are respiratory complications and vitamin B6‐dependent seizures.^4^ In a study from 2019,^4^ patients observed with manifestations of vitamin B6‐dependent seizures had a 100% mortality rate. In HPP, these seizures are considered a sign of impending death.^4^

**TABLE 1:** Clinical subtypes of hypophosphatasia^3^

Type	Bone symptoms	Dental symptoms
Perinatal (severe)	Results in stillbirth or death within days/weeks after birth Disturbances of the Ca^2+^/PO₄^3−^ metabolism, seizures, hypomineralization, secondary severe lung hypoplasia	
Perinatal (benign)	Prenatal findings of limb shortening and bowing of long bones Postnatal spontaneous improvement of skeletal defects	
Infantile—first symptoms appear within the first 6 months of life	Hypomineralization, rickets, premature craniosynostosis, hydrocephalus, irritability, seizures, muscular hypotonia, nephrocalcinosis	
Childhood/juvenile—first symptom present after the first year of life	Rickets, short stature, failure to thrive, delayed walking, muscular hypotonia, chronic pain in the upper extremities, gastrointestinal problems	Premature loss of teeth Dental caries
Adult	Osteomalacia, chondrocalcinosis, osteoarthropathy, low bone mineral density, osteoporosis, stress and fragility fractures, delayed fracture healing, arthritis, chronic inflammatory condition, renal function abnormalities	Sudden loss of teeth
Odontohypophosphatasia		Premature loss of teeth Dental caries

The subtypes are inherited in different manners: the severe perinatal and the infantile subtypes are inherited in an autosomal recessive way, whereas the remaining subtypes can be inherited in both an autosomal dominant and recessive manner, with the dominant being the most frequent mode of inheritance. To study the cellular pathophysiology of HPP and classify variants, studies of the residual activity of the deficient enzymes have been conducted. These indicate that severe variants cause low residual activities whereas milder variants leave the enzyme with some residual activity.^5^

The treatment of HPP depends on the clinical presentation. Pre 2015, symptomatic treatment was the only option, which involved dietary measures with lowering of calcium intake, orthopedic surgery, reduction of hypercalciuria, pain management with NSAID, and treatment of seizures with vitamin B6.^6^ However, in 2015^7^ an enzyme replacement treatment was approved for pediatric use in some jurisdictions. The active component of the treatment is asfotase alfa, a human fusion protein consisting of a TNSALP iso domain, an IgG Fc domain, and a deca‐aspartate peptide used as a bone‐targeting domain. The TNSALP iso domain works as a substitute for the deficient TNSALP and thus reduces the extracellular accumulations of TNSALP substrates.^7^

Asfotase alfa is currently only used to treat pediatric‐onset HPP (https://alexion.com/our-medicines/medicines/strensiq). The therapeutic effects include improved bone mineralization causing increased osteoid thickness and bone mineral density. Furthermore, a significant improvement of gross motor, fine motor, and cognitive development in age‐equivalent scores are also seen. Patients typically also experience rapid and sustained improvements in physical function, pain levels, and the ability to participate in daily activities.^6^ Plasma PLP levels are significantly reduced, which is an indication of a successful TNSALP substitution.^6^, ^8^ Treatment with asfotase alfa also improves rib cage mineralization of patients requiring respiratory assistance.^6^, ^8^

## 2 CASE REPORT

A female patient was born at 40 weeks' gestation with a birth weight of 3300 g, length of 54 cm, and head circumference of 33 cm. She was the first child of unrelated parents, with no family history of HPP. There were no abnormal findings on prenatal imaging. In the first months after birth, the patient presented with excessive crying, irritability, increased tone in the legs, vomiting, and failure to thrive. Hip dysplasia and metaphyseal changes were detected on X‐rays and the dysplasia was successfully corrected with Dennis Brown hip abduction orthosis. A urine metabolic screening revealed raised phosphoethanolamine levels (15 times the upper limit, also in plasma) and further investigations showed low ALP at 44 U/L (lower reference at 132 U/L) and slight hypercalcemia. These results led to a suspicion of childhood HPP and at 13 months the diagnosis was confirmed by sequencing of *ALPL*, which identified homozygosity for a missense variant (NM_000478.6): c.542C>T (p.Ser181Leu), reported previously in 2001.^9^ The patient also presented with periods of cyanosis, hypertonia, unconsciousness, and abnormal eye movements leading to treatment with valproate, since epilepsy was suspected. An EEG was normal. She was changed to Keppra and given vitamin B6 after the diagnosis of HPP with some effect. At age 2 years, she presented with temper tantrum‐like episodes, which stopped after iron therapy was introduced at age 3 years.

Through childhood, the patient had difficulties eating due to nausea and vomiting, and at age 4 years tube feeding was initiated. She had the feeding tube until the age of 9 years. She suffered from chronic skeletal pain from early childhood, notably affecting the long bones, requiring daily use of analgesics. On the visual analog scale (VAS), a scale used to determine pain intensity experienced by an individual, the patient stated an average score of 7 (with a score of 0 indicating no pain, and a score of 10 indicating maximum pain) at age 13 years, causing difficulties participating in exercise and outdoor activities as well as social isolation. Additionally, the pain caused sleeping problems, which added to her pains and made the patient stay home from school multiple days a week. On X‐rays and ultrasound, the patient presented with age‐appropriate bone maturation with no deformity or rickets, normal hips, and normal joints. She had no difficulties walking and did not need a walker nor a cane. She was, however, given a wheelchair for longer distances, due to limited endurance. The patient had premature loss of teeth, and at age 3 years she had lost almost all her primary teeth. The orthodontic examination revealed no craniosynostosis but showed classic symptoms of HPP: loose teeth and enlarged periodontal spaces. She also presented with insufficient mastication, and prostheses were made to correct this.

Treatment with asfotase alfa was considered for a long time, primarily related to the lack of reimbursement, particularly with the lack of radiographic abnormalities. However, in the light of persistently high pain VAS scores with an established pain phenotype severely affecting her life and because blood analysis showed significantly raised P‐pyridoxal‐5‐phosphate at >2500 mmol/L (ref. 15‐75 mmol/L) supporting the association between HPP and pains, reimbursement for asfotase alfa treatment was given.

At the baseline examination at age 13 years, the patient presented with a height of 143.3 cm and a weight of 38.2 kg. Laboratory results showed low ALP, raised P‐PEA levels, and significantly raised levels of P‐pyroxidal‐5‐phosphate (Table 2). Serum calcium, phosphate, 25‐hydroxy vitamin D, thyroid and parathyroid hormones were normal. Bone mineral density (BMD) measured by Dual‐Energy X‐ray Absorptiometry (DEXA) scan showed normal BMD Z‐score in both the lumbar spine and total body. Ultrasound of the kidneys showed a few hyperechogenic areas, but neither Ca^2+^ deposits, hydronephrosis nor nephrocalcinosis were detected. X‐ray of the thorax showed a normal skeleton with no signs of rickets. Ophthalmologic examination revealed normal vision but with hypermetropia. The patient was premenarchal at the time of initiation of treatment but with onset 6 months into treatment.

**TABLE 2:** Laboratory results at baseline before treatment and during follow‐up after 3, 6, 12, and 18 months

Laboratory studies	Baseline (13 years)	3 months	6 months	12 months	18 months
S‐ALPL (U/L) (ref. 90‐250 U/L)	20	5570	> 6000	>6000	>6000
P‐PLP (mmol/L) (ref. 20‐122 mmol/L)	>2500	41	26	54	94
U‐PEA (μM) (ref. 0‐3.5 μM)	11	N.a.	3	0	N.a.
Total body BMD, Z‐score	+0.5	N.a.	N.a.	+0.4	+0.3
Total body BMC (kg)	1.45	N.a.	N.a.	1.56	1.54
VAS score (average) (ref. 0‐10)	7	4	2	2	3
Height (cm)	143.3 (−2 SD)		144.7 (<2 SD)	145.3 (<2 SD)	

Abbreviations: ALPL, alkaline phosphatase; BMC, bone mineral content; BMD, bone mineral density; n.a.; not available; PEA, phosphoethanolamine; PLP, P‐pyridoxal‐5‐phosphate.

Asfotase alfa treatment was started at 1 mg/kg (40 mg/mL) given subcutaneously 6 times per week. After 12 months the regimen was changed to three weekly injections of 2 mg/kg (80 mg/mL) due to skin reactions and psychological difficulties concerning the injection. Following this change, the patient expressed a significant improvement concerning her psychological reaction to the injections, but also more days with pain. The patient agreed that a reduction in skeletal pain was of greater importance than fewer injections and agreed to change back to the six weekly injections. She is now using a subcutaneous cannula, that is used for 6 days, further helping her psychologically.

After 3 months of treatment, the patient exhibited marked clinical improvement in her chronic skeletal pain with an average VAS‐score of 3, and analgesia use dropped from on average daily use to 1 in 4 days. During treatment, her VAS‐score and analgesics use continued to decline. The patient expressed a notable difference in her overall well‐being, she was more sociable and participated in activities with her peers. The treatment did not seem to affect the patient's height as she only grew 2 cm in 18 months. Dentition was not affected by the treatment—her teeth remained loose and with large periodontal spaces; however, no loss of permanent teeth was seen.

Biochemical studies during treatment showed a reduction in P‐PLP and P‐PEA (Table 2), whereas the results of P‐ALP were uninterpretable as this was now a measurement of exogenous ALP. Her values of calcium, phosphate, PTH, and D‐vitamin stayed normal.

## 3 DISCUSSION

This case suggests an association between children with HPP without radiographic signs of skeletal involvement, but with a debilitating pain phenotype, and a significant pain reduction on enzyme replacement therapy. Thus, this therapy should be considered in such patients.

The typical patient with HPP presents with skeletal symptoms such as low bone mineral density, deformities of the bones and rickets in children and osteomalacia in adults. In the case reported here, no significant radiographic signs were seen. When the patient was diagnosed with childhood HPP at 13 months, metaphyseal changes were detected on X‐ray. This was possibly a sign of residual undetected severe intrauterine changes that had disappeared at the time of birth, which could indicate that the patient had the perinatal type of HPP instead of childhood HPP. Thus, it is possible that at some point before birth the patient had significant radiographic signs. The lack of specific skeletal symptoms meant that the main factors to monitor during treatment were her skeletal pain and biochemical values. The cause of skeletal pain is not completely known, but it may be caused by accumulation of PPi and PLP in joints and ligaments, which can induce inflammation and pain. To investigate this explanation, an examination of the patient's synovial fluid was tried but failed, making the reason behind the pain still unclear. However, the pain reduction is most probably caused by asfotase alfa mimicking TNSALP and thus hydrolyzing PPi and PLP, lowering the accumulation of these in the joints and ligaments. This decrease is reflected in the biochemical results. The treatment did not seem to affect her growth significantly. An X‐ray from 2014 (before treatment) showed non‐fused epiphyseal plates. Her bone age was estimated at 13 years, while her chronological bone age was 10 years. On an X‐ray from 2016, around the start of asfotase alfa treatment, almost completely fused epiphyseal plates were seen, indicating little to no potential for the treatment affecting her growth. When the dosage regimen was changed from 6 times weekly to 3 weekly injections, the patient expressed more days with pain. The explanation could be a short T½ of the medication; an average half‐life of 2.28 ± 0.58 days with a range of 0.74 to 9.94 days has been detected, based on a population pharmacokinetic analysis (https://alexion.com/Documents/Canada/Strensiq-Product-Monograph_English_13July2016.aspx, February 20, 2020). This could indicate that our patient metabolizes the medicine more rapidly than the average person taking part in the clinical trials. To keep a therapeutic asfotase alfa concentration in the patient, a more frequent injection regimen was required.

As mentioned earlier, the *ALPL* variant identified in the patient reported here has been reported previously (in the paper named S164L).^9^ The variant was discovered by Sanger sequencing of the *ALPL* gene in a 15‐year‐old girl. The proband, who was heterozygous for the variant, presented with bowing of arms and legs and disproportionate short stature, but with no serious illness and no severe radiographic signs. Serum ALPL was in the subnormal range (116 U/L) and bone biopsy showed disturbance of mineralization. There was no family history of neither short stature nor bone disease. The residual ALPL activity resulting from the variant was evaluated by transfection of COS‐1 cells with both mutated and wild‐type plasmids. The results indicated that the S164L substitution had little or no effect on the wild‐type monomer, that is, did not exert a dominant‐negative effect, and was likely recessively inherited, hence the patient must have had another undetected variant besides the S164L variant. This is in accordance with the identification of the variant in homozygous form in the patient reported here. The proband and the patient of our case report also share similarities regarding the lack of notable radiographic signs but a pain phenotype.

In a case report published in 2017,^10^ a 12‐month treatment with asfotase alfa of a 16‐year‐old male patient with severe childhood HPP is described. Before treatment, the patient presented with significant skeletal manifestations, chronic skeletal pain requiring almost daily use of analgesics, and impaired physical function. After 3 months of treatment, the patient experienced significant improvement in skeletal pain along with less use of analgesics. After 6 months, the patient was able to participate in outdoor activities. This patient and our patient share the same debilitating pain phenotype affecting everyday activities. Both patients experienced a decrease in analgesic‐use along with improvement of skeletal pain after initiating the asfotase alfa treatment. An assessment^11^ from 2015 based on the NCT00952484 and NCT01203826 clinical trials, reported the response to 5 years of treatment with asfotase alfa. The 13 patients included in the study were all between the ages of 6 to 12 years with a diagnosis of either infantile HPP or childhood HPP. Before treatment, 45% of the patients reported limited activities due to bone pain and 39% of patients reported a need for analgesics. At baseline, Pediatric Outcome Data Collection Instrument (PODCI) global function median score was at 27.0 (range: −2.0, 55.0). The normative mean score for a healthy population is 50 SD ± 10, with a range of −77 (worst) to 58 (best).^11^ The PODCI score indicates how much pain interferes with normal activities, with a high score indicating little to no interference. Thus, a decrease in PODCI score is desired. After 5 years of treatment, the median score had improved to 52 (range: 28.0, 57.0). The median parent‐reported Child Health Assessment Questionnaire (CHAQ) pain was elevated before treatment at 20.0 (range: 0.0, 72.0). The score is based on a 0 to 3 point system (0 indicating no pain to 3 max pain) applied to 24 questions. The points are added together to create an overall score with a range from 0 (best) to 72 (worst). A CHAQ pain score of 0 is preferred.^11^ After 5 years of treatment, the median was at 0 (range: 0.0, 60.0), indicating less to no pain for most patients included in the study. Overall, children in this study experienced rapid and sustained improvement in physical functions, pain levels, and the ability to participate in sports activity during the asfotase alfa treatment.

The case report, the clinical study assessment, and our case report demonstrate the likely effect of asfotase alfa therapy on functional gains and chronic skeletal pain in patients diagnosed with childhood HPP. Thus, an association between asfotase alfa and an improvement of a debilitating pain phenotype is likely. Asfotase alfa therapy should be considered in this patient group.

## CONFLICT OF INTEREST

The authors declare no potential conflict of interests.

## AUTHOR CONTRIBUTIONS

Olivia Sarah Strandbech: drafting of the manuscript. Allan Lund: provided analyzed and interpreted data, clinical assessment of the patient, revising of the manuscript. Elsebet Østergaard: critical revising of the manuscript, approved the final version.

## INFORMED CONSENT

All procedures followed were in accordance with the ethical standards of the responsible committee on human experimentation (institutional and national) and with the Helsinki Declaration of 1975, as revised in 2000. Informed consent from the patient's parents was obtained.

## ANIMAL RIGHTS

This article does not contain any studies with human or animal subjects performed by any of the authors.

## GUARANTOR AUTHOR

Elsebet Østergaard is the guarantor author of this article.

## ETHICS STATEMENT

Ethical approval was not required for this case report.

## Data Availability

Patient data are saved in the electronic patient records of the hospital.

## References

[1] Komaru K , Ishida‐Okumura Y , Numa‐Kinjoh N , Hasegawa T , Oda K . Molecular and cellular basis of hypophosphatasia. J Oral Biosci. 2019;61:141‐148. 31400546 10.1016/j.job.2019.07.003

[2] Orimo H . The mechanism of mineralization and the role of alkaline phosphatase in health and disease. J Nippon Med Sch. 2010;77:4‐12. 20154452 10.1272/jnms.77.4

[3] Hofmann C , Girschick HJ , Mentrup B , et al. Clinical aspects of hypophosphatasia: an update. Clin Rev Bone Mineral Metabol. 2013;11:60‐70.

[4] Whyte MP , Leung E , Wilcox WR , et al. Natural history of perinatal and infantile hypophosphatasia: a retrospective study. J Pediatr. 2019;209:116‐24.e4. 30979546 10.1016/j.jpeds.2019.01.049

[5] Mornet E . Genetics of hypophosphatasia. Arch Pediatr. 2017;24:5s51‐5s56. 29405932 10.1016/S0929-693X(18)30014-9

[6] Scott LJ . Asfotase alfa: a review in paediatric‐onset hypophosphatasia. Drugs. 2016;76:255‐262. 26744272 10.1007/s40265-015-0535-2

[7] Choida V , Bubbear JS . Update on the management of hypophosphatasia. Ther Adv Musculoskelet Dis. 2019;11:1759720X19863997. 10.1177/1759720X19863997. PMC6676257 31413732

[8] Whyte MP , Greenberg CR , Salman NJ , et al. Enzyme‐replacement therapy in life‐threatening Hypophosphatasia. New Engl J Med. 2012;366:904‐913. 22397652 10.1056/NEJMoa1106173

[9] Lia‐Baldini AS , Muller F , Taillandier A , et al. A molecular approach to dominance in hypophosphatasia. Hum Genet. 2001;109:99‐108. 11479741 10.1007/s004390100546

[10] Bowden SA , Adler BH . Asfotase alfa treatment for 1 year in a 16 year‐old male with severe childhood hypophosphatasia. Osteoporos Int. 2018;29:511‐515. 29046930 10.1007/s00198-017-4267-x

[11] Whyte MP , Madson KL , Phillips D , et al. Asfotase alfa therapy for children with hypophosphatasia. JCI Insight. 2016;1:e85971. 27699270 10.1172/jci.insight.85971 PMC5033855

